# Protective Effect of Theaflavin on Erythrocytes Subjected to *In Vitro* Oxidative Stress

**DOI:** 10.1155/2013/649759

**Published:** 2013-12-21

**Authors:** Mahejabeen Fatima, Rajesh Kumar Kesharwani, Krishna Misra, Syed Ibrahim Rizvi

**Affiliations:** ^1^Department of Biochemistry, University of Allahabad, Allahabad 211002, India; ^2^Division of Applied Science & Indo-Russian Center for Biotechnology (IRCB), Indian Institute of Information Technology, Allahabad 211012, India

## Abstract

Antioxidant and free radical scavenging effect of black tea theaflavins has been shown in many epidemiological studies. In the present work we report the protective mechanism of tea theaflavins on biomarkers of oxidative stress, which are elevated during stress conditions. We hereby report the *in vitro* effect of theaflavins on erythrocyte malondialdehyde (MDA), intracellular reduced glutathione (GSH), and plasma membrane redox system (PMRS) of rats. The effect of theaflavin on PMRS has also been validated through an *in silico* docking simulation study using Molegro Virtual Docker (MVD). We report that theaflavins show significant protection to erythrocyte against oxidative stress induced by *tert*-butyl hydroperoxide (t-BHP). The findings suggest a possible protective role of theaflavins as antioxidant.

## 1. Introduction

**Figure 1:**
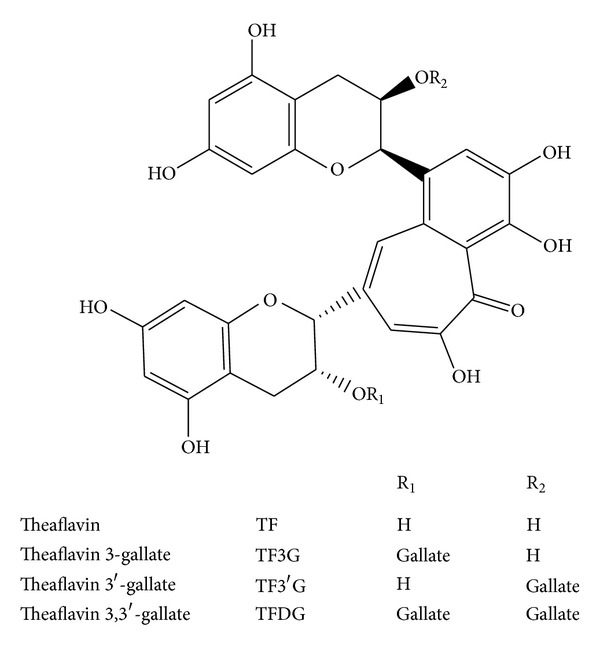
Polyphenols of black tea.

Tea is one of the ancient and most popularly consumed beverages worldwide. Mostly it is consumed as green and black tea. Among the types of tea, black tea accounts for approximately 80% of the global production. More research has been done on green tea, but in recent years black tea has attracted significant attention for its extensive health promotional effect [1]. Black tea contains 30–40% polyphenols which include theaflavins (TFs), thearubigins (TRs), and bisflavonols. Theaflavins are mixture of theaflavin 3-gallate (TF3G), theaflavin 3′-gallate (TF3′G), and theaflavin 3,3′-digallate (TFDG), and the common feature of all these polyphenols is the presence of a seven-member benztropolone ring (Figure 1). Epidemiological studies and associated clinical observations indicate that black tea polyphenols possess many health beneficial properties including antioxidant, antimutagenic, anticancer, antipathogenic anti-inflammatory, and anticlastogenic effects [2–7]. Reports show that black tea consumption may reduce the risk of several diseases like cardiovascular disease, diabetes, osteoporosis, and neurodegenerative disease [8–11]. The exact mechanism underlying these properties remains speculative.

Oxidative stress has been shown to be coupled with altered homeostasis that leads to imbalance between oxidant production and/or antioxidant levels thus causing damage to biomolecules, dysregulation of normal physiology/metabolism coupled with neurodegeneration, cognitive impairment, immunosuppression, and ageing [12, 13]. The potential role of free radicals, reactive oxygen species, and antioxidants in the etiology of chronic diseases has stimulated extensive research in current years [14].

In mammalian system, erythrocytes have enzymatic as well as nonenzymatic antioxidant defenses such as glutathione (GSH) and vitamin E to recognize the levels of ROS and to limit the damage they impose [15]. Eukaryotic cells also display an entirely distinct electron export system known as plasma membrane redox system (PMRS) which is an electron transport chain in the plasma membrane by which cells oxidize electron donors, typically NADH and/or NADPH, and transfer the resulting electrons to the extracellular acceptors. These defenses decline with age, and during the disease conditions, some reports show that the decline in antioxidant defence can be augmented by supplementation of antioxidants [16–18]. The PMRS has three major entities: the intracellular electron donor species, oxidoreductases and electron carrier proteins, and extracellular electron acceptors. The cytochrome b5 reductase (EC 1.6.2.2) is an important enzyme of PMRS in erythrocyte.

In recent years, antioxidants have been subjected to numerous epidemiological studies that had related their consumption to reduction in the occurrence of oxidative damage related diseases. Therefore, much interest has been focused mainly on the use of natural antioxidants for the human health improvement [19].

The present study was undertaken to determine the antioxidant effect of tea theaflavins on biomarkers of oxidative stress in erythrocytes of rat. The present study reports a protective effect of theaflavins on oxidation induced alteration in malondialdehyde (MDA) level, intracellular reduced glutathione (GSH) content, and plasma membrane redox system (PMRS) activity on rat erythrocytes subjected to increased oxidative stress by incubating with *tert*-butyl hydroperoxide (*t*-BHP). We also report the *in silico* docking of theaflavin with cytochrome b5 reductase which is the constituent of PMRS. The docking of theaflavin has been compared with FAD, NADPH, NADH, epigallocatechin gallate, catechin, epicatechin, quercetin, and resveratrol.

## 2. Material and Methods

### 2.1. Experimental Animals

The study was carried out on normal adult (10–12 months) Wistar rats of average body weight of 180–200 gram. The protocol of study was in accordance with the guidelines of the Institutional Ethical Committee. Rats were purchased from the animal house of the Indian Institute of Toxicological Research (IITR). Experimental rats were allowed to acclimatize before the commencement of the experiment. They were kept in plastic cages in the animal house, maintained under standardized conditions, and allowed standard pelleted animal food and water ad libitum.

The whole blood was collected by cardiac puncture into syringes containing heparin. The blood was centrifuged at 800 ×g for 10 minutes at 4°C. The RBCs were washed twice with cold phosphate buffer saline (PBS) (0.9% NaCl, 10 mM Na_2_HPO_4_, and pH 7.4) after removal of plasma, buffy coat, and upper 15% of cells.

### 2.2. Measurements of Lipid Peroxidation

The method of Esterbauer and Cheeseman was used to measure erythrocyte malondialdehyde (MDA) [20]. PRBCs (0.2 mL) were suspended in 3 mL PBS containing 0.5 mM glucose. The suspension (0.2 mL) was added to 1 mL of 10% trichloroacetic acid (TCA) and 2 mL of 0.67% thiobarbituric acid (TBA), boiled for 20 minutes at a temperature >90°C, and cooled and the absorbance read at 532 nm. Concentration of MDA is calculated using extinction coefficient (*ε* = 15,600 M^−1^ cm^−1^). The concentration of MDA is expressed as nmol·mL^−1^ of packed erythrocytes.

### 2.3. Measurements of Intracellular Glutathione

Erythrocyte GSH was measured following the method of Beutler [21]. The method was based on the ability of the –SH group to reduce 5,5′-dithiobis,2-nitrobenzoic acid (DTNB) and form a yellow coloured anionic product whose absorbance is measured at 412 nm. Concentration of GSH is expressed in mg·mL^−1^ packed RBCs and was calculated from standard plot.

### 2.4. Induction of Oxidative Stress

Oxidative stress was induced *in vitro* by incubating washed erythrocytes with *tert*-butyl hydroperoxide (*t*-BHP; 10^−5^ mol·L^−1^ final concentration) with or without theaflavin in the above experiments.

### 2.5. *In Vitro* Incubation of Erythrocyte with Theaflavin

The effect of theaflavin on the following parameters, erythrocyte MDA content, GSH status, and PMRS activity, was investigated as follows. A stock solution (10 mM) of theaflavin (Sigma Aldrich, St. Louis, USA, cat number T5550-1MG) was prepared in ascorbic acid; further dilutions (10^−4^ M–10^−6^ M) were done with PBS. The concentration of ascorbic acid was always <0.01% (w/v) in the final solution. The *in vitro* effect of theaflavin was evaluated by incubating erythrocytes with theaflavin at different doses (10^−5^ M–10^−7^ M final concentration) for 30 minutes at 37°C. The erythrocytes were again washed three times with PBS, pH 7.4, to remove any amount of the compound and finally PRBCs were used for the assay of MDA, GSH, and PMRS activity. In parallel control experiments, PRBCs were incubated with equal amount of solvent.

### 2.6. Measurement of Erythrocyte PMRS Activity

The activity of the erythrocyte plasma membrane redox system (PMRS) was measured by following the reduction of ferricyanide according to the method of Avron and Shavit [22]. PRBCs (0.2 mL) were suspended in PBS containing 5 mM glucose and 1 mM freshly prepared potassium ferricyanide to a final volume of 2.0 mL. The suspensions were incubated for 30 min at 37°C and then centrifuged at 1,800 ×g at 4°C and supernatant was collected. The supernatant was assayed for ferrocyanide content using 4,7-diphenyl-1,10-phenanthroline disulfonic acid disodium salt and measuring absorption at 535 nm (*ε* = 20,500 M^−1^·cm^−1^). The results are expressed in *μ*mol ferrocyanide/mL PRBC/30 min. The dose-dependent effects of theaflavin on erythrocyte PMRS were studied.

### 2.7. Protein and Ligands Structure Preparation

For the docking simulation study, 3-dimensional coordinates of Human NADH-cytochrome b5 reductase were downloaded from the Protein Data Bank bearing id 1UMK with resolution 1.75 Å [23, 24]. The downloaded protein contains 275 residues together with FAD as a bound ligand and 753 water molecules of crystallization. During the preparation of receptor protein for docking simulation input file, all the heteroatoms were removed by using Molegro Virtual Docker (MVD). The missing explicit hydrogen atom and bond order information was assigned. The potential binding site cavity has been selected suggested by kesharwani et al., 2012 [25]. The structure of theaflavin was downloaded from PubChem database and prepared by using MVD.

### 2.8. Docking Simulation

After complete preparation of receptor and ligand, the docking simulation study was performed by automated docking software MVD on HP Z800 workstation. It is fast and robust docking software. The docking simulation iteration has been specified up to 2000 for each ligand. The simulation results return five different poses and are evaluated on the basis of MoleDock and H-bond score [26].

### 2.9. Statistical Analysis

Statistical analysis was done by the software Graph Pad Prism 5 version 5.01. The experimental results are expressed as mean ± standard deviation of triplicate measurements. To assess relationship between parameters, one-way analysis of variance, ANOVA, was done with significant value (*P* < 0.05) in Bonferroni's multiple comparison test.

## 3. Results and Discussion

The current study was designed to determine the effect of theaflavin on the biomarkers of oxidative stress: MDA, GSH, and PMRS. These biomarkers were preferred because they all have been implicated in the potential pathways linking oxidation to pathologic processes. Under oxidative stress, erythrocyte membranes are vulnerable to lipid peroxidation that involves cleavage of polyunsaturated fatty acids at their double bonds leading to the formation of aldehydes, MDA, and 4-hydroxynonenal (4-HNE) [27, 28]. These byproducts may influence the properties of cell membranes and their physiological functions such as cellular deformity, fluidity, permeability, and alteration of structural and functional integrity of cells [29]. These products may be mutagenic and carcinogenic and consequently implicated in the pathophysiology of numerous diseases including diabetes [30], cancer [31], and lung diseases [32].

**Figure 2:**
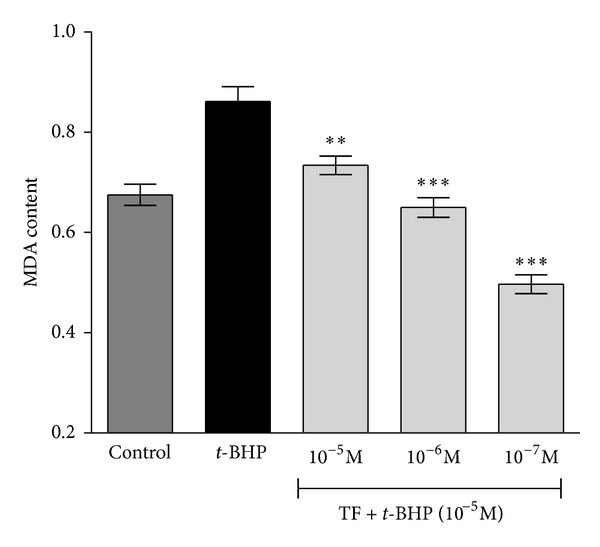
Concentration-dependent effect of black tea theaflavins on *tert*-butyl hydroperoxide- (*t*-BHP-) induced changes in malondialdehyde (MDA) content in normal Wistar rat erythrocytes. The effect of theaflavins was evaluated on erythrocytes subjected to oxidative stress by incubation with *t*-BHP (10^−5^ mol/L). The MDA content is expressed as nmol/mL packed erythrocytes. **P < 0.05; ***P < 0.001 as compared with *t*-BHP treated.

In the present study, MDA content increased above basal level significantly in erythrocytes subjected to increased oxidative stress by incubating them with *t*-BHP. The presence of black tea theaflavin in the incubation medium protected the erythrocyte from *t*-BHP-induced oxidative stress, as evidenced by decrease in level of MDA. A significant (*P* < 0.05) protective effect of theaflavins was observed at concentration of 10^−7^ M (Figure 2).

Glutathione, an efficient thiol containing antioxidant present in almost all living cells, is also considered as a sensitive biomarker of cell functionality and viability. The physiological function of GSH is multifaceted and implicated in the cellular defense against hydroperoxides and free radicals [33] and thus provides a first line of defense. Its depletion is associated with increased sensitivity to oxidative stress and susceptibility to numerous diseases including cancer, cardiovascular diseases, and neurodegenerative diseases [34]. In addition, GSH also provides reducing capacity in several reactions such as reduction of dehydroascorbate to ascorbate and formation of deoxyribonucleotides by ribonucleotide reductase.

**Figure 3:**
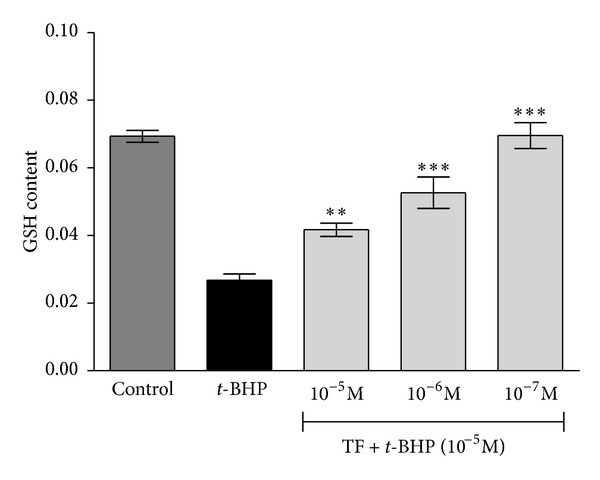
Concentration-dependent effect of black tea theaflavins on *tert*-butyl hydroperoxide- (*t*-BHP-) induced changes in intracellular reduced glutathione (GSH) content in normal Wistar rat erythrocytes. The effect of theaflavins was evaluated on erythrocytes subjected to oxidative stress by incubation with *t*-BHP (10^−5^ mol/L). Significant increase was observed in level of GSH. GSH content is expressed as mg/mL PRBCs. **P < 0.05; ***P < 0.001 as compared with *t*-BHP treated.

Induction of oxidative stress following incubation with *t*-BHP resulted in a decrease in GSH content in erythrocytes. Theaflavin demonstrated a significant (*P* < 0.05) protective effect against *t*-BHP-induced GSH oxidation, as evidenced by increase in GSH level at concentration of 10^−7^ M (Figure 3). No effect was observed with ascorbic acid (at concentration equivalent to its role as solvent of theaflavin) on erythrocyte MDA or GSH.

The observation suggests that, during the process of oxidative damage, theaflavin may act as exogenous antioxidant and could donate a proton to a radical, thereby forming a relatively stable product. In the previous studies theaflavin has been shown to be a potent *in vitro* antioxidant [35]. These antioxidative capacities of theaflavin may be attributed to the presence of antioxidant pharmacophores within the molecule that have the optimal configuration for free radical scavenging, that is, an *ortho*-3′4′-dihydroxyl (catechol) group or 3′4′5′-trihydroxyl (gallate) group in the B ring, a gallate group esterified at the 3 position of the C ring, and hydroxyl groups at 5 and 7 positions of the A ring [36]. The free radical scavenger ability of a compound is partly related to its standard one-electron reduction potential (E°′), a measure of the reactivity of an antioxidant as electron or hydrogen donor under standardized conditions. Studies have revealed that the lower E°′ indicates that less energy is required for the donation of electron or hydrogen and is an important factor in determining antioxidant activity. Tea theaflavins have E°′ values higher than ascorbate (vitamin C) which is a superior antioxidant or hydrogen donor compared to tea polyphenols [12, 37]. Researches have shown that TFDG possesses higher antioxidative activity due to greater number of hydroxyl (OH) groups as compared to catechins, considered to be necessary for exerting radical scavenging ability [37]. Perhaps an additional group increases the total number of phenyl hydroxyl groups and makes the gallate containing TF more able to donate proton due to the resonance delocalization.

**Figure 4:**
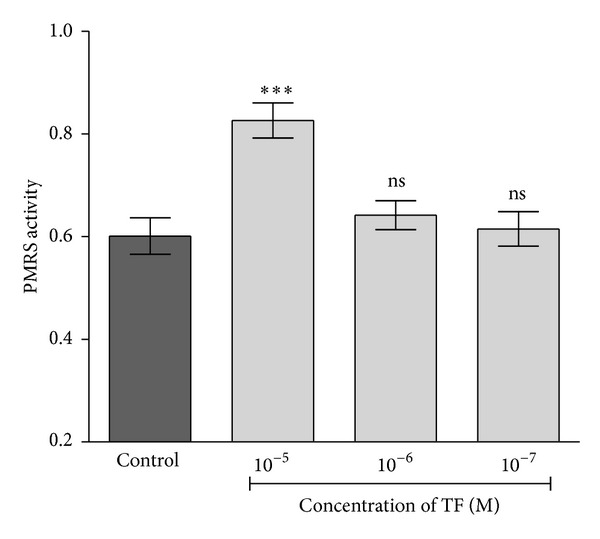
Dose-responsive effect of theaflavin on PMRS in erythrocytes isolated from rat blood. PMRS activity expressed in terms of micromole ferrocyanide/mL PRBC/30 min. ***P < 0.001 as compared with control.

Erythrocyte PMRS plays an important role in the regulation of antioxidant status [16, 17]. Incubation of tea theaflavin for 30 min at 10 *μ*M final concentration significantly (*P* < 0.05) activated erythrocyte PMRS activity (Figure 4); ascorbic acid alone 0.01% (w/v) did not show any effect on PMRS activity. However, the effect of theaflavin at 1 *μ*M and 0.1 *μ*M (final concentration) was not significant. In the present study, the activation of the erythrocyte PMRS activity by theaflavin suggests that it can donate electrons to the PMRS for the reduction of extracellular oxidants and for the recycling of ascorbate. The ability of theaflavin to activate the erythrocyte PMRS may provide protection against oxidative changes and also in diseased conditions. Erythrocyte PMRS provides the cell with an additional level of defense against extracellular oxidants and enables the cell to respond to changes in the intra- and extracellular redox milieu [17]. It has been shown that the electron donating ability of polyphenols depends on the position and degree of hydroxylation [36, 37]. Perhaps, an additional group in theaflavin increases the total number of phenyl hydroxyl groups and makes the gallate containing TF more able to donate a proton due to the resonance delocalization [37]. These hydrogen atoms may possibly be transferred to ROS to neutralize their harmful effects. The activation of erythrocyte PMRS by theaflavin is an interesting finding and needs more study to determine the exact mechanism of action.

**Figure 5:**
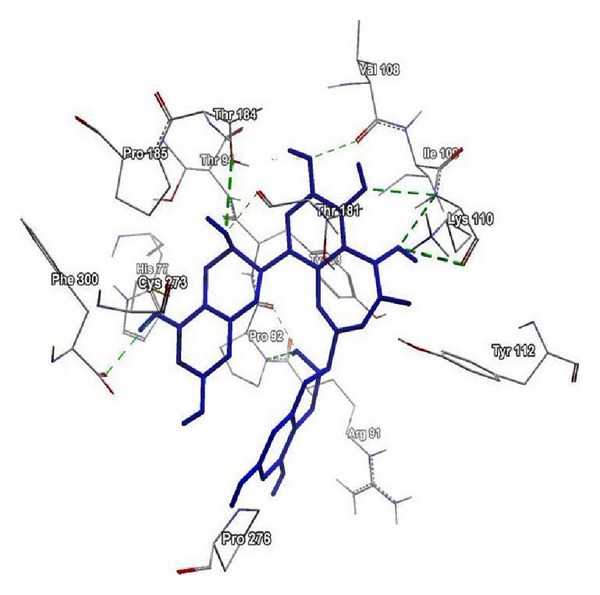
Low energy bound conformation of theaflavin in hydrogen bonding view with interacting amino acids of Human NADH-cytochrome b5 reductase protein at the active site cavity.

The comparative docking simulation result of theaflavin and selected polyphenols with Human NADH-cytochrome b5 reductase is shown in Table 1 and presented in terms of MoleDock Score and H-bonding energy [25]. Computational methods are now being used for designing and interpretation of hypothesis-driven experiments providing validation of experimental data. Our results show that theaflavin has a favourable MoleDock Score and H-bonding energy thus providing binding affinity for Human NADH-cytochrome b5 reductase. The formation of hydrogen bonds provides additional force to stabilize the ligand-protein complex required for the activity of the Human NADH-cytochrome b5 reductase (Figure 5). We have reported the *in silico* binding affinity of EGCG, epicatechin, quercetin, catechin, and resveratrol [25]; however, our observations show that theaflavin has a better binding affinity for Human NADH-cytochrome b5 reductase when compared with other polyphenols.

A number of the biological effects of theaflavin have been attributed to its antioxidative property, although the exact mechanism in many cases is not completely explained. It has been shown in animal models and humans that consumption of black tea increases the plasma antioxidant capacity significantly thus protecting RBCs against oxidative damage induced by ROS and RNS [38]. Moreover, a number of human studies involving black tea reveal a significant increase in plasma antioxidant capacity in humans ~1 hour after consumption of moderate amounts of tea 1–6 cups per day [6]. Studies regarding bioavailability have reported that the maximum plasma concentration of theaflavin was 1 ng/mL in volunteers after oral intake of 700 mg of TFs (equivalent to 30 cups of black tea) [39]. Black tea polyphenols may suppress free radicals and protect cells against H_2_O_2_-induced damage [40]. TFs are capable of anticancerous action by inducing apoptosis and also act as efficient inhibitor of antiapoptotic proteins (Bcl-2 family proteins) [41]. Other metabolic effects of black tea have been also reported in improving the plasma lipid profiles in animals after high fat diet [42], reducing blood glucose and blood triglyceride levels in aged rats and antioxidative effect leading to alcohol intoxication by normalization of cellular metabolism through modulation of the antioxidative defense enzymes [43].

In conclusion, the present study provides evidence for the strong antioxidant effect of theaflavin which it can exert in human system providing an efficient protection against conditions which result in oxidative stress. The activation of erythrocyte PMRS by theaflavin is an interesting finding in view of the speculation that activation of PMRS may be an antiaging strategy [44]. The *in silico* docking simulation of theaflavin with Human NADH-cytochrome b5 reductase validates the present observation of PMRS activation. The findings emphasize the need for more research on black tea compounds. Questions regarding bioavailability, metabolism, and evaluation corresponding to the optimal amount of consumption of black tea are not completely explained so far.

## Figures and Tables

**Table 1:** Comparative docking simulation result of selected polyphenols with Human NADH-cytochrome b5 reductase together with FAD, ligand from X-ray crystallized data of protein data bank (1 umk·pdb) using Molegro Virtual Docker (MVD) [25].

Serial number	Ligands	MoleDock Score	H-bonding energy
1	FAD	−232.638	−20.532
2	NADPH	−209.954	−13.985
3	beta-NADH	−208.235	−13.506
4	Theaflavin	−153.946	−13.580
5	EGCG	−131.595	−9.012
6	Quercetin	−113.611	−10.033
7	Catechin	−110.472	−9.063
8	Epicatechin	−102.952	−14.638
9	Resveratrol	−102.074	−10.272
